# Evaluation on the Potential for Hepatotoxic Components from Herba Epimedii to Induce Apoptosis in HepG2 Cells and the Analysis of the Influence of Metabolism in Liver Microsomes

**DOI:** 10.3390/molecules29061354

**Published:** 2024-03-19

**Authors:** Lin Zhang, Cai Zhang, Xiyi Peng, Zhaojuan Guo, Song Yang, Dongjun Fu

**Affiliations:** 1 Beijing Research Institute of Chinese Medicine, Beijing University of Chinese Medicine, Beijing 100013, China; zhangcai6789@sina.com (C.Z.); zhaojuanguo@bucm.edu.cn (Z.G.); fengyasong_100@163.com (S.Y.); fudongjun@bucm.edu.cn (D.F.); 2 School of Traditional Chinese Medicine, Beijing University of Chinese Medicine, Beijing 100013, China; xixi0808ya@163.com

**Keywords:** Herba Epimedii, hepatotoxicity, apoptosis, metabolism, liver microsomes

## Abstract

The potential hepatotoxicity of Herba Epimedii is a focal point in traditional Chinese medicine security applications. As determined in our previous study, the flavonoid constituents of Herba Epimedii, sagittatoside A, icariside I, baohuoside I and icaritin, are related to the hepatotoxicity of this herb. However, the hepatotoxic mechanism of these components needs to be clarified further, and whether these components can maintain their injury action following liver metabolism needs to be confirmed. Herein, the effects of sagittatoside A, icariside I, baohuoside I and icaritin on the apoptosis of HepG2 cells and the expression of key proteins, including Bax, Bcl-2, Caspase-3 and Caspase-9, were evaluated. Moreover, with liver microsome incubation, the influences of metabolism on the apoptotic activities of these components were investigated. Then, by HPLC–MS/MS analyses, the in vitro metabolic stability of these components was determined after incubation with different kinds of liver microsomes to explain the reason for the influence. The results suggested that sagittatoside A, baohuoside I and icaritin could induce apoptosis, which is likely to be closely related to the induction of the intrinsic apoptosis pathway. After metabolic incubation, the sagittatoside A and icaritin metabolism mixture could still induce apoptosis due to less metabolic elimination, while the icariside I and baohuoside I metabolism mixtures respectively got and lost the ability to induce apoptosis, probably due to quick metabolism and metabolic transformation. The findings of this study may provide important references to explore the material basis and mechanism of the hepatotoxicity of Herba Epimedii.

## 1. Introduction

Herba Epimedii is a common yang-strengthening herbal medicine that is widely used in clinical traditional Chinese medicine (TCM) for various treatments and to promote health care. As its range of applications is continuously expanding, an increasing number of adverse events have been reported with the use of Herba Epimedii, notably liver injury [1,2]. The national system database for monitoring adverse drug reactions (ADRs) included 124 liver injury cases related to Herba Epimedii from 2012 to 2016, and among them, 89 cases meet the diagnostic criteria of biochemical indexes in the guidelines for the diagnosis and treatment of drug-induced liver injury [1]. Similar to other TCMs, the components of Herba Epimedii are characterized as having various types of complex structures, mainly flavonoids, lignins, sesquiterpenes, ionones, phenol glycosides, phenylethanoid glycosides and other compounds [3,4]. Mounting evidence has suggested that the prenylated flavonoids in Herba Epimedii are not only the major biologically active compounds but are also most closely related to hepatotoxicity. To examine the toxicity of icariin, epimedin A, epimedin B, epimedin C, sagittatoside A, sagittatoside B, 2″-*O*-rhamnosylicariside II and baohuoside I, zebrafish embryos were exposed to these compounds to simulate the liver. Zebrafish embryos have hepatocytes from 2 days after fertilization and exhibited concordance in the metabolic pathway compared to the traditional models [5,6]. After observing the morphology of the zebrafish larvae and recording their mortality, the results suggested that the order of toxicity of these compounds was 2″-*O*-rhamnosylicariside II and sagittatoside B > epimedin C and sagittatoside A > baohuoside I, icariin, epimedin A, and epimedin B [7]. Some studies have illustrated that icariside I and baohuoside I can exacerbate NLRP3 inflammasome activation triggered by ATP and nigericin. This caused increases in alanine aminotransferase (ALT) and aspartate aminotransferase (AST) levels, hepatocyte necrosis and hepatic inflammation after the administration of a nonhepatotoxic dose of lipopolysaccharide (LPS), and led to wild-type (WT) C57BL/6 mice being susceptible to DILI. These data indicated that both icariside I and baohuoside I could contribute to liver injury due to enhanced NLRP3 inflammasome activation and might be responsible for the hepatotoxicity of Herba Epimedii [8,9]. Furthermore, baohuoside I was found to be intrinsically toxic to HL7702 and HepG2 cells and cause liver injury in female ICR mice. It was considered that the corresponding mechanism was that baohuoside I could repress farnesoid X receptor (FXR) transcriptional activity by targeting the estrogen receptor α (ERα) for degradation, known to disrupt bile acid (BA) homeostasis and induce BA accumulation to ultimately induce liver injury [10]. Nevertheless, in view of the TCM characteristics of multiple components and multiple targets, elucidating the hepatotoxic components in Herba Epimedii still awaits further confirmation.

**Figure 1:**
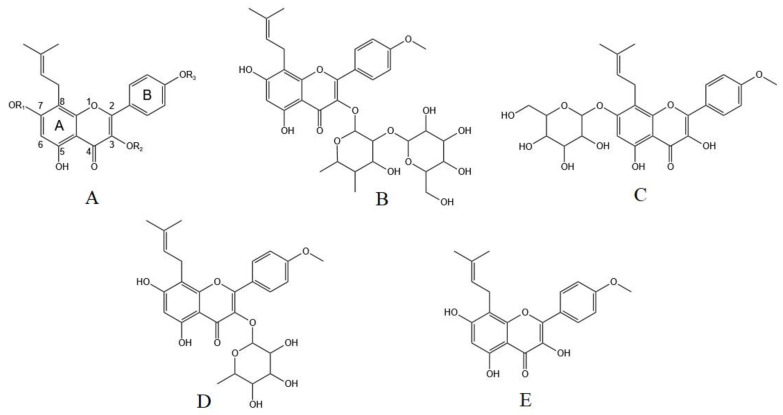
Chemical structures of the potential hepatotoxic components of Herba Epimedii. (**A**) prenylated flavonoid mother nucleus; (**B**) sagittatoside A; (**C**) icariside I; (**D**) baohuoside I; and (**E**) icaritin.

In our previous studies, based on high-performance liquid chromatography–tandem mass spectrometry (HPLC–MS/MS) analysis and cytotoxic evaluations in HL7702 cells and HepG2 cells, sagittatoside A (CAS registry number 118525-35-2), icariside I (CAS registry number 56725-99-6), baohuoside I (CAS registry number 113558-15-9) and icaritin (CAS registry number 118525-40-9) could be important potential hepatotoxic components in Herba Epimedii extracts [11]. These components share the same prenylated flavonoid core structure but differ in terms of their glycosyl substitutions at the C-3 and C-7 positions (Figure 1). Moreover, we further found that treating HL-7702 cells and HepG2 cells with certain concentrations of these components could cause various increases in AST, lactate dehydrogenase (LDH) release, malondialdehyde (MDA) activity and (or) intracellular reactive oxygen species (ROS) fluorescence intensity and could cause decreases in glutathione (GSH) activity and/or mitochondrial membrane potential (MMP) to different extents [11,12,13]. These correlated biological indicators were investigated, and the hepatotoxic mechanisms of the screened components were inferred to be associated with the induction of apoptosis. However, the direct apoptotic effect of these potential hepatotoxic compounds has not been confirmed. In addition, as the main organ responsible for drug metabolism, the liver contains many kinds of drug-metabolizing enzymes and determines the types of drug metabolism and clearance, which ultimately affect the role of the drug following oral consumption. Therefore, the metabolic stability of these potential hepatotoxic components needs to be explored, and the effect of metabolism on their ability to induce apoptosis must be determined.

In this study, the apoptosis of HepG2 cells induced by sagittatoside A, icariside I, baohuoside I and icaritin was evaluated to explore the hepatotoxic mechanism, and the expression of key proteins associated with apoptosis was detected by flow cytometry and western blotting. Furthermore, the ability of these components to induce apoptosis after incubation with human liver microsomes was evaluated again, reflecting the influences of metabolism on apoptosis. Finally, the in vitro metabolism of these potential hepatotoxic compounds was analyzed by incubation with liver microsomes from different species and HPLC–MS/MS detection. Subsequently, the change in the percentage of the compound remaining over time and half-life (*T*_1/2_) were calculated to determine whether the original form of the compound could be retained to produce hepatotoxicity. The above data are very important for determining the key material basis and mechanism for the hepatotoxicity of Herba Epimedii.

## 2. Results

### 2.1. HepG2 Cell Apoptosis Induced by Hepatotoxic Compounds

**Figure 2:**
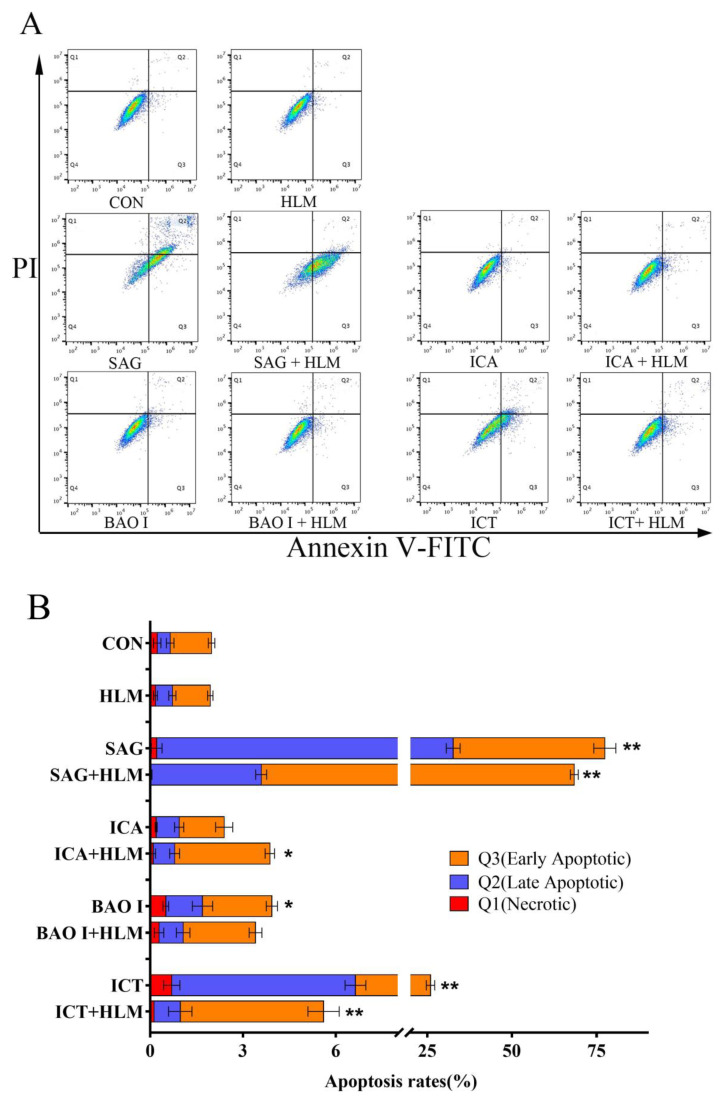
Induction of apoptosis after HepG2 cells were treated with different concentrations of the hepatotoxic components of Herba Epimedii. (**A**) Dot plots constructed from the flow cytometry fluorescence intensity measurements and flow cytometric analysis of Annexin V-FITC and PI fluorescence in cells treated with each hepatotoxic compound for 24 h and stained with Annexin V-FITC and PI. The upper left (Q1), upper right (Q2) and lower right (Q3) quadrants were used to determine the percentages of apoptotic cells. (**B**) Bar graph summarizing the apoptosis rates in each treatment group. Values are presented as the mean ± standard deviation of triplicate experiments (*n* = 3). * *p* < 0.05 and ** *p* < 0.01 compared with the control group. CON: control; HLM: human liver microsomes; SAG: sagittatoside A; ICA: icariside I; BAO I: baohuoside I; ICT: icaritin.

After 24 h of treatment with each hepatotoxic compound, HepG2 cell apoptosis was assessed by flow cytometry. The dot plots constructed after double staining with Annexin V-FITC and PI and the apoptosis assay results are shown in Figure 2. The apoptosis rate of the cells treated with the middle and high concentrations of sagittatoside A was 77.44 ± 1.22%, indicating significant apoptosis (*p* < 0.01). Icariside I did not cause a change in the apoptosis rate (2.39 ± 0.34%) at the tested concentration. The apoptosis rate of the cells treated with baohuoside I was 3.93 ± 0.20% (*p* < 0.05). The apoptosis rate of the cells treated with icaritin was 25.93 ± 0.66% (*p* < 0.01). Overall, these results suggested that sagittatoside A, baohuoside I and icaritin induced HepG2 cell apoptosis, which was mainly consistent with the results from the early apoptosis assessment.

### 2.2. Influence of the Hepatotoxic Compounds on Bax, Bcl-2, Caspase-9 and Caspase-3 Expression

**Figure 3:**
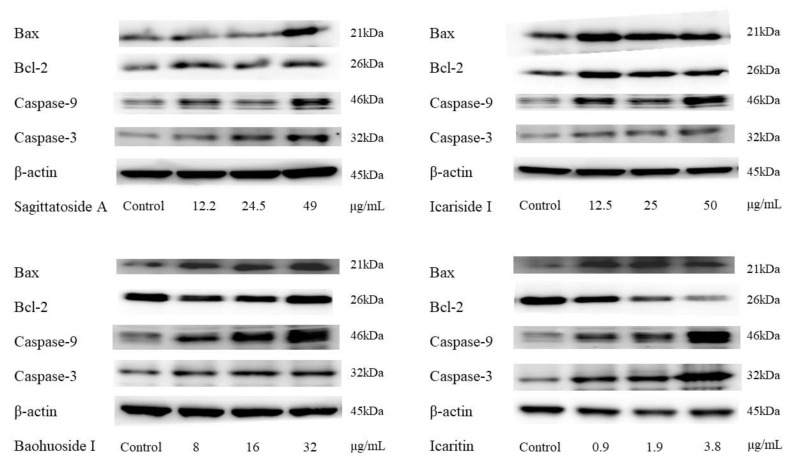
Western blot analysis of the expression of Bax, Bcl-2, caspase-9 and caspase-3 in HepG2 cells induced by the hepatotoxic components of Herba Epimedii after 24 h of culture.

**Figure 4:**
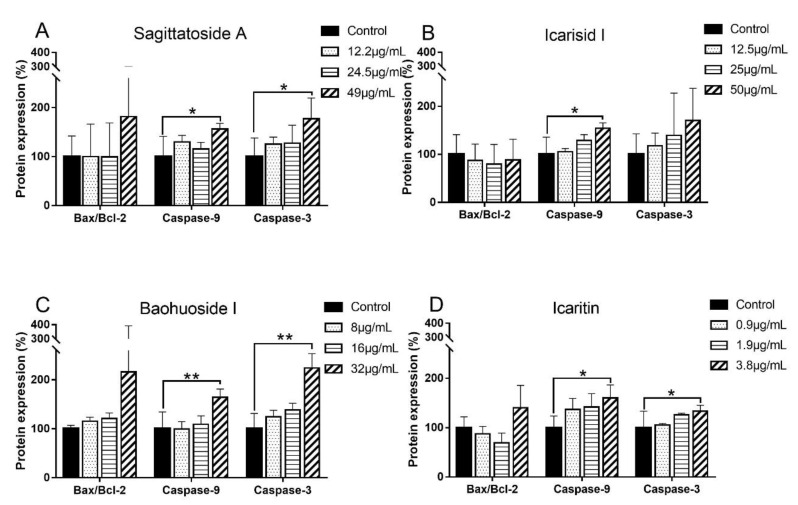
Relative expression levels of caspase-3 and caspase-9 and the ratios of Bax/Bcl-2 in HepG2 cells induced by different concentrations of sagittatoside A (**A**), icariside I (**B**), baohuoside I (**C**) and icaritin (**D**). Values are presented as the mean ± standard deviation of triplicate experiments (*n* = 3). * *p* < 0.05 and ** *p* < 0.01 compared with the control group.

The association between HepG2 cell apoptosis induced by each hepatotoxic compound and the expression of apoptotic factors was then investigated. The protein expression levels of the apoptosis-associated factors Bcl-2, Bax, caspase-9 and caspase-3 were evaluated by western blot analysis. The expression of these proteins in HepG2 cells treated with the hepatotoxic compounds and the protein expression levels are displayed in Figure 3 and Figure 4. As shown in Figure 3, after 24 h of culture in the presence of different concentrations of sagittatoside A, baohuoside I and icaritin, the expression of Bax, caspase-9 and caspase-3 in HepG2 cells displayed increasing trends in a concentration-dependent manner, while the expression of Bcl-2 displayed a decreasing trend with increasing icaritin concentration. Further quantitative analysis showed that at high concentrations, sagittatoside A increased the Bax/Bcl-2 ratio and caused a significant increase (*p* < 0.05) in caspase-9 and caspase-3 expression compared with that in untreated cells (Figure 4A). High concentrations of icariside I caused a significant increase (*p* < 0.05) in caspase-9 expression only (Figure 4B). Additionally, high concentrations of baohuoside I increased the Bax/Bcl-2 ratio and caused a significant increase (*p* < 0.01) in caspase-9 and caspase-3 expression (Figure 4C). Moreover, high concentrations of icaritin also increased the Bax/Bcl-2 ratio and caused a significant increase (*p* < 0.05) in caspase-9 and caspase-3 expression (Figure 4D). These results further confirmed that, to a certain extent, the mechanism by which sagittatoside A, baohuoside I and icaritin induce hepatotoxicity is through apoptosis.

### 2.3. HepG2 Cell Apoptosis Induced by Hepatotoxic Compounds after Metabolism

The mixture obtained through each hepatotoxic compound metabolized with human liver microsomes was applied to cells for 24 h, and HepG2 cell apoptosis was assessed by flow cytometry. The dot plots constructed after double staining with Annexin V-FITC and PI and the apoptosis assay results are shown in Figure 2. The apoptosis rate of the cells treated with the sagittatoside A metabolism mixture was 68.42 ± 1.10%, indicating significant apoptosis (*p* < 0.01). The apoptosis rate of the cells treated with the mixture of icariside I metabolism was 3.86 ± 0.22% and caused a change in the apoptosis rate (*p* < 0.05). The apoptosis rate of the cells treated with the baohuoside I metabolism mixture was 3.40 ± 0.19%, which was insignificant. The apoptosis rates of the cells treated with the middle and high concentrations of icaritin were 5.61 ± 0.83% (*p* < 0.01). Overall, these results suggested that through metabolism, the induction of cell apoptosis by sagittatoside A and icaritin was weakened but could be maintained. Due to metabolism, baohuoside I lost the ability to induce cell apoptosis, while icariside I maintained this ability.

### 2.4. Metabolic Stability of the Hepatotoxic Compounds

**Figure 5:**
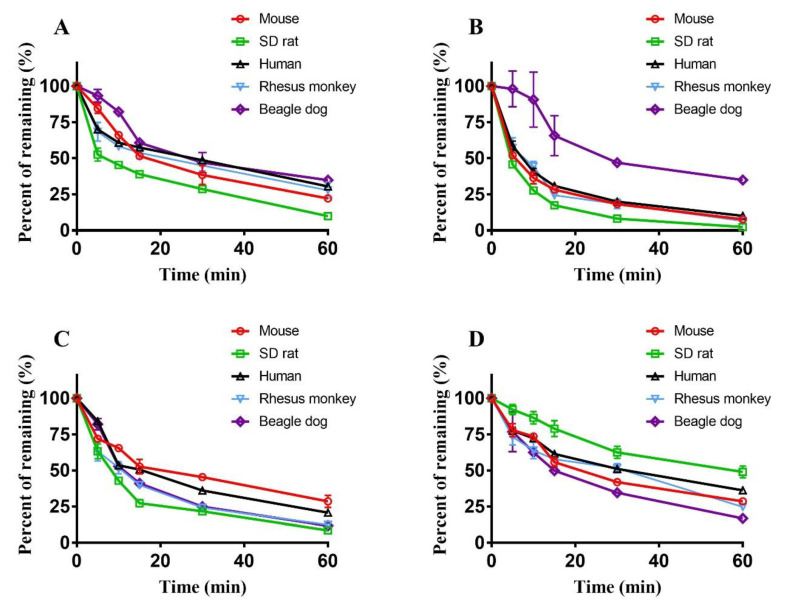
Phase I elimination curves of sagittatoside A (**A**), icariside I (**B**), baohuoside I (**C**) and icaritin (**D**) in liver microsomes from different species (*n* = 2).

The phase I metabolism elimination curves of sagittatoside A, icariside I, baohuoside I and icaritin in liver microsomes from different species are shown in Figure 5, and the corresponding metabolic parameters are displayed in Table 1. The depletion of these compounds followed first-order kinetics under the experimental conditions, and significant species differences were observed. After incubation for 60 min, the amounts of sagittatoside A remaining in the liver microsomes of mice, SD rats, humans, rhesus monkeys and beagle dogs were 22.20 ± 0.36%, 9.94 ± 1.92%, 30.49 ± 0.15%, 27.76 ± 5.49%, and 34.80 ± 0.35%, respectively, and the calculated in vitro *T*_1/2_ values were *T*_1/2_ (SD rats) < *T*_1/2_ (mice) < *T*_1/2_ (rhesus monkeys) < *T*_1/2_ (beagle dogs) < *T*_1/2_ (humans). The amounts of icariside I in the liver microsomes of mice, SD rats, humans, rhesus monkeys and beagle dogs were 7.69 ± 1.17%, 2.38 ± 0.32%, 10.08 ± 0.43%, 6.50 ± 0.49%, and 34.93 ± 2.83%, respectively, and the *T*_1/2_ values were *T*_1/2_ (SD rats) < *T*_1/2_ (rhesus monkeys) < *T*_1/2_ (mice) < *T*_1/2_ (humans) < *T*_1/2_ (beagle dogs). The amounts of baohuoside I remaining in the liver microsomes of mice, SD rats, humans, rhesus monkeys and beagle dogs were 28.58 ± 4.12%, 8.52 ± 0.15%, 20.74 ± 0.39%, 12.41 ± 1.02%, and 11.74 ± 0.78%, respectively, and the *T*_1/2_ values were *T*_1/2_ (SD rats) < *T*_1/2_ (beagle dogs) < *T*_1/2_ (rhesus monkeys) < *T*_1/2_ (humans) < *T*_1/2_ (mice). The amounts of icaritin remaining in the liver microsomes of mice, SD rats, humans, rhesus monkeys and beagle dogs were 28.55 ± 0.04%, 48.95 ± 4.13%, 36.23 ± 1.12%, 24.73 ± 2.38%, and 16.75 ± 0.20%, respectively, and the *T*_1/2_ values were *T*_1/2_ (beagle dogs) < *T*_1/2_ (rhesus monkeys) < *T*_1/2_ (mice) < *T*_1/2_ (humans) < *T*_1/2_ (SD rats). The predicted CLint_(liver, in vivo)_ value of each compound presented the same trend: CLint_(liver, in vivo)_ (humans) < CLint_(liver, in vivo)_ (beagle dogs) < CLint_(liver, in vivo)_ (rhesus monkeys) < CLint_(liver, in vivo)_ (SD rats) < CLint_(liver, in vivo)_ (mice).

**Figure 6:**
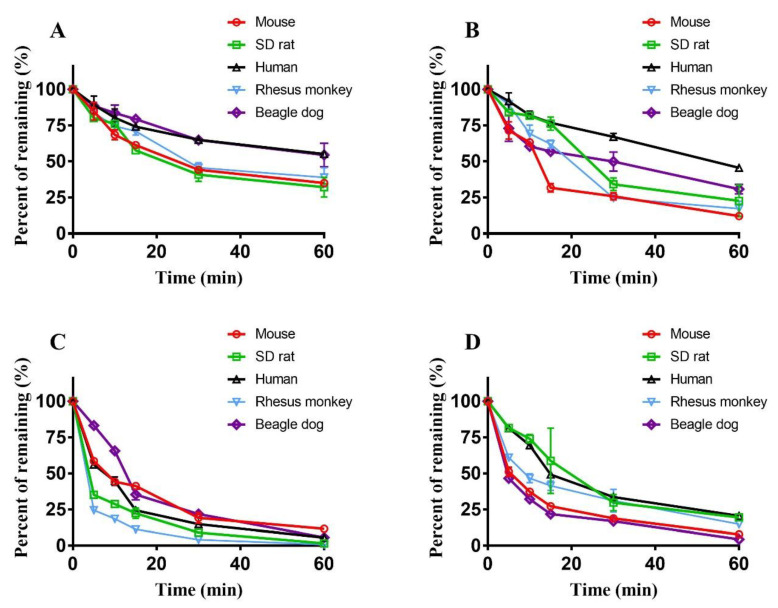
Phase II elimination curves of sagittatoside A (**A**), icariside I (**B**), baohuoside I (**C**) and icaritin (**D**) in liver microsomes of different species (*n* = 2).

The phase II metabolism elimination curves of the hepatotoxic components in liver microsomes from different species are shown in Figure 6, and the corresponding metabolic parameters are displayed in Table 2. The depletion of these compounds also followed first-order kinetics and had species differences. The final amounts of sagittatoside A remaining after phase II metabolism in the liver microsomes of mice, SD rats, humans, rhesus monkeys and beagle dogs were 35.01 ± 2.13%, 32.12 ± 6.79%, 55.17 ± 0.08%, 38.92 ± 6.69%, and 54.43 ± 8.12%, respectively, and the *T*_1/2_ values were *T*_1/2_ (SD rats) < *T*_1/2_ (rhesus monkeys) < *T*_1/2_ (mice) < *T*_1/2_ (beagle dogs) < *T*_1/2_ (humans). The amounts of icariside I remaining in the liver microsomes of mice, SD rats, humans, rhesus monkeys and beagle dogs were 12.18 ± 0.64%, 22.71 ± 10.94%, 45.62 ± 1.52%, 17.23 ± 5.20%, and 30.79 ± 3.26%, respectively, and the *T*_1/2_ values were *T*_1/2_ (mice) < *T*_1/2_ (rhesus monkeys) < *T*_1/2_ (SD rats) < *T*_1/2_ (beagle dogs) < *T*_1/2_ (humans). The amounts of baohuoside I remaining in the liver microsomes of mice, SD rats, humans, rhesus monkeys and beagle dogs were 11.64 ± 1.63%, 1.55 ± 0.33%, 5.56 ± 0.60%, 0.86 ± 0.17%, and 5.69 ± 0.33%, respectively, and the *T*_1/2_ values were *T*_1/2_ (rhesus monkeys) < *T*_1/2_ (SD rats) < *T*_1/2_ (beagle dogs) < *T*_1/2_ (humans) < *T*_1/2_ (mice). The amounts of icaritin remaining in the liver microsomes of mice, SD rats, humans, rhesus monkeys and beagle dogs were 7.73 ± 0.17%, 19.44 ± 0.36%, 20.51 ± 0.97%, 14.86 ± 0.76%, and 4.42 ± 0.67%, respectively, and the *T*_1/2_ values were *T*_1/2_ (beagle dogs) < *T*_1/2_ (mice) < *T*_1/2_ (SD rats) < *T*_1/2_ (rhesus monkeys) < *T*_1/2_ (human). Comparison of the data from phase II metabolism with those from phase I metabolism indicated that the *T*_1/2_ values and amounts of sagittatoside A and icariside I remaining after phase II metabolism in the liver microsomes of each species were higher. The corresponding values of baohuoside I and icaritin were lower after phase II metabolism than after phase I metabolism. The predicted phase II metabolism CLint_(liver, in vivo)_ of each compound in the different kinds of liver microsomes also followed the order Clint_(liver, in vivo)_ (humans) < Clint_(liver, in vivo)_ (beagle dogs) < Clint_(liver, in vivo)_ (rhesus monkeys) < Clint_(liver, in vivo)_ (SD rats) < Clint_(liver, in vivo)_ (mice).

## 3. Materials and Methods

### 3.1. Chemicals and Reagents

Baohuoside I and hesperidin (internal standard (IS), CAS registry number 520-26-3) were obtained from the National Institute for the Control of Pharmaceutical and Biological Products (Beijing, China). Sagittatoside A, icariside I and icaritin were purchased from Yuanye Bio-Technology Co., Ltd. (Shanghai, China). Phase I and II metabolic stability kits (separately equipped with mouse, Sprague–Dawley (SD) rat, human, rhesus monkey or beagle dog liver microsomes, 20 mg protein/mL) were obtained from IPhase Biosciences (Beijing, China). HepG2 cells were purchased from China Infrastructure of Cell Line Resources (Beijing, China). The BD Pharmingen™ Annexin V FITC apoptosis detection kit was purchased from BD Biosciences (San Jose, CA, USA). A Caspase-3 rabbit polyclonal antibody (19677-1-AP-100 µL) and Caspase-9/p35/p10 rabbit polyclonal antibody (10380-1-AP-100 µL) were purchased from Proteintech Group, Inc. (Rosemont, IL, USA). Furthermore, anti-Bcl-2 (1:2000, ab196495), anti-Bax (1:1000, ab32503), goat anti-rabbit IgG H&L (HRP, 1:5000, ab6721) and goat anti-mouse IgG H&L (HRP, 1:5000, ab6789) antibodies were purchased from Abcam (Cambridge, UK). β-Actin (1:5000, #8457) was purchased from Cell Signaling Technology, Inc. (Boston, MA, USA). All organic solvents and reagents were HPLC grade. Deionized water was prepared using a Milli-Q laboratory water purification system (Millipore, Bedford, MA, USA).

### 3.2. Apoptosis Analysis by Flow Cytometry

HepG2 cells were cultured in Roswell Park Memorial Institute (RPMI) 1640 medium (GIBCO, Grand Island, NY, USA) supplemented with 10% heat-inactivated fetal bovine serum (PAN Biotech GmbH, Aidenbach, Germany), 100 U/mL penicillin, and 100 μg/mL streptomycin. The cells were maintained in a humidified atmosphere with 5% CO_2_ at 37 °C. Upon reaching 90% confluence, the cells were prepared as single-cell suspensions at a density of 1 × 10^4^ cells/mL by treatment with 0.25% trypsin with 0.02% EDTA and then dispensed into 12-well plates (Corning, NY, USA) with a total volume of 1.5 mL per well. After culturing for 24 h, the medium was discarded, and the cells were incubated with fresh medium or medium containing different hepatotoxic compounds at various concentrations for an additional 24 h. Based on the IC_50_ value of the compound [11,12], the concentrations of sagittatoside A, icariside I, baohuoside I and icaritin to treat cells were set to 49, 50, 32 and 3.8 μg/mL, respectively. After treatment, the cells were collected for apoptosis analysis.

To investigate the effects of the hepatotoxic compounds on HepG2 cell apoptosis, flow cytometry was performed with a FACSCalibur flow cytometer (BD Biosciences) using an Annexin V-FITC apoptosis detection kit. The cells were prepared according to the manufacturer’s instructions. Briefly, the collected cell pellets were washed three times by centrifugation at 2000 rpm and 4 °C for 5 min with PBS and resuspended in binding buffer. Then, the cells were incubated with Annexin V-FITC for 15 min, propidium iodide (PI) was added, and the cells were incubated for another 5 min. The whole labeling process was performed in the dark. After fluorescent labeling, the cell suspension was immediately analyzed by flow cytometry. Cells in the early phases of apoptosis can bind Annexin V-FITC but exclude PI, while cells in late apoptosis or those that are necrotic can be stained with Annexin V-FITC and PI simultaneously.

### 3.3. Western Blot Analysis

To explore the mechanism of apoptosis induced by the hepatotoxic compounds, the expression of key proteins related to apoptosis, including Bax, Bcl-2, Caspase-3 and Caspase-9, was assessed by western blotting. HepG2 cells were treated with different concentrations of the hepatotoxic compounds for 24 h and collected with a cell scraper. The concentrations of sagittatoside A were set to 12.2, 24.5, and 49 μg/mL; those of icariside I were 12.5, 25, and 50 μg/mL; baohuoside I was used at 8, 16, and 32 μg/mL; and icaritin was tested at 0.9, 1.9 and 3.8 μg/mL. The cells were washed twice with PBS and lysed in total protein cell lysis buffer. Protein concentrations were determined using a BCA protein assay kit (Thermo Fisher Scientific, Waltham, MA, USA). Then, to destroy the 3-dimensional protein structure, the proteins in the loading buffer were heated at 95 °C for 5 min. An equal amount of protein from each sample was separated by SDS-PAGE on 10% SDS–polyacrylamide gels. After the protein was transferred to a polyvinylidene difluoride membrane (Millipore, Bedford, MA, USA), the membrane was incubated in blocking buffer (5% nonfat dairy milk in TBST) for 2 h and probed with various antibodies (1:1000) overnight at 4 °C. The membrane was then probed with a secondary antibody (1:5000) for 2 h at room temperature, and detection was performed with an enhanced chemiluminescence kit (Millipore, Bedford, MA, USA). β-Actin was used as the control to confirm equal loading. Densitometry index analysis of the bands was conducted using a gel imagery system. The changes in the protein expression levels are expressed as percentages relative to the levels measured in the corresponding control groups.

### 3.4. Apoptosis Analysis after Liver Microsomes Incubation

The incubation mixtures were prepared following the instructions from the manufacturer of the metabolic stability kits and contained sagittatoside A, icariside I, baohuoside I, and icaritin, of which the final concentrations were 49, 50, 32 and 3.8 μg/mL. The final concentration of organic solvents in the incubation mixture was 1% (*v*/*v*). After preincubation for 5 min at 37 °C, metabolic reactions of phase I and II were both initiated by the addition of human liver microsomes (final concentration, 0.20 mg protein/mL). Phase I metabolism is mediated by the enzyme cytochrome P450 (CYP), and phase II metabolism is mediated by UDP-glucuronosyltransferase (UGT). The whole reaction process was performed under aerobic conditions. After 30 min, an equal volume of cold acetonitrile was added and vortexed for 1 min to terminate the reactions. Subsequently, 200 μL of the resulting solution was centrifuged at 3000 rpm and 4 °C for 10 min. Then, 100 μL of supernatant was dried at 40 °C with nitrogen gas, and the residue was redissolved in 100 μL with fresh medium to prepare for treating cells. Negative controls (incubation without substrate) were prepared in the same manner.

### 3.5. Incubation with Liver Microsomes for Metabolic Stability Analysis

The incubation mixtures were also prepared following Section 2.4 and contained sagittatoside A, icariside I, baohuoside I, and icaritin, of which the final concentrations were 5.00 μM. After the mixtures were incubated separately in phase I or phase II metabolism systems for 5, 10, 15, 20, 30, 45 and 60 min, the reactions were terminated by adding an equal volume of cold acetonitrile (with 7 μM IS) and vortex mixing for 1 min. Subsequently, 200 μL of the resulting solution was centrifuged at 3000 rpm and 4 °C for 10 min. Then, 100 μL of supernatant was dried at 40 °C with nitrogen gas, and the residue was redissolved in 100 μL of 50% methanol to prepare the samples for HPLC–MS/MS detection. Time zero samples (prepared by first adding cold acetonitrile and then adding NADPH for phase I metabolism or NADPH and UDPGA for phase II metabolism) and negative controls (incubation without substrate) were prepared separately in the same manner.

### 3.6. Metabolism Analysis by HPLC–MS/MS

HPLC analysis was performed on an AB Sciex ExionLC HPLC system (Santa Clara, CA, USA) equipped with an autosampler, a vacuum degasser unit, a quaternary pump, a diode array detector (DAD) and a column compartment. Samples were separated on an ACQUITY ΜPLC BEH C_18_ column (2.1 × 100 mm, 1.7 μm) connected to an ACQUITY ΜPLC BEH C_18_ precolumn (2.1 × 5 mm, 1.7 μm) at 40 °C. The mobile phases were water with 0.05% formic acid (A) and methanol-acetonitrile (*v*/*v* 1:1) with 0.05% formic acid (B), and the elution gradient was as follows: 70% A (0.00–1.00 min), 2% A (2.50–3.50 min), and 70% A (3.51–7.00 min). The flow rate was set at 0.23 mL/min, and the injection volume was 5 μL.

Electrospray ionization–mass spectrometry (ESI–MS) analyses were performed on an AB Sciex QTRAP 5500 (EG250911902) mass spectrometer (Santa Clara, CA, USA). Detection was performed with multiple reaction monitoring (MRM) in ESI negative ion mode. Specific ion pairs and the parameters of the compounds are summarized in Table 3. The spray voltage was set to −4500 V, the curtain gas was 25 psi, the collision gas was set to medium, both auxiliary gases 1 and 2 were used at 45 psi, and the heating temperature was 550 °C.

In accordance with the above HPLC–MS/MS analysis method, the content of each compound in the sample at each time point was measured. Then, using the substrate depletion method, the content of each compound in the time zero sample was set as 100%, and the percent residual substrate was calculated. The relationship between the natural logarithmic value of the percent residual substrate and time point was explored by linear regression using GraphPad Prism 5.0 software, and the slope (−*k*) was obtained. The in vitro elimination *T*_1/2_, intrinsic clearance (CLint_(mic)_), liver clearance in vitro (CLint_(liver, in vitro)_) and predicted liver clearance in vivo (CLint_(liver, in vitro)_) were calculated using the following equations [14]:

*T_1_*_/2_ = −0.693/*k* (1)

CLint_(mic)_ = −k/mg microsome protein per mL (2)

CLint_(liver, in vitro)_ = CLint_(mic)_ ×(45 mg microsomes/gm liver) × (gm liver/kg b.w.) (3)

CLint_(liver, in vitro)_ = CLint_(liver, in vitro)_ × hepatic blood flow/(CLint_(liver, in vitro)_ + hepatic blood flow) (4)

The liver weights of the mice, SD rats, humans, rhesus monkeys and beagle dogs were 88, 40, 20, 30, and 32 g/kg, respectively, and the hepatic blood flow rates of the mice, SD rats, humans, rhesus monkeys and beagle dogs were 90, 55, 21, 44, and 31 mL/min/kg, respectively [15].

### 3.7. Statistical Analysis

Each set of results shown is representative of three separate experiments. Statistical analyses were performed with the SPSS software suite, version 17.0. All data are expressed as the mean ± standard deviation of the mean. Statistical analysis was performed using one-way ANOVA followed by the Bonferroni correction for individual comparisons between the means of multiple groups and corresponding control groups. A value of *p* < 0.05 was considered to indicate statistical significance.

## 4. Discussion

Apoptosis is a key feature of several acute and chronic liver diseases and underlies DILI [16,17,18]. Cell apoptosis in the liver is initiated by two distinct pathways: the extrinsic pathway, which is activated by death receptors located at the cellular membrane, and the intrinsic pathway (also called the mitochondrial pathway), which involves mitochondria [19]. The B-cell lymphoma 2 (Bcl-2) family of proteins, which are generally bound to the mitochondrial membrane, can regulate activation of the mitochondrial pathway. The Bcl-2 family has proapoptotic members, such as the Bcl-2-associated X (Bax) and Bcl-2-associated agonist of cell death (Bad), and antiapoptotic members, such as Bcl-2 and myeloid cell leukemia-1 (Mcl-1). The relative expression of proapoptotic and antiapoptotic Bcl-2 proteins in the outer membrane of the mitochondria, which can be indicated by factors such as the ratio of Bax/Bcl-2, play an important role in the regulation of the mitochondrial apoptotic pathway [20] and could determine the release of cytochrome c from the mitochondria. The release of cytochrome c is a central event in apoptosis, and once cytochrome c enters the cytosol and binds to cytosolic apoptotic protease-activating factor 1 (Apaf-1), a caspase cascade reaction occurs [21]. This results in the activation of caspase-9 and processing of the main apoptosis executioner caspase-3, thus reaching the “point of no return” [22]. In our previous studies, we assumed that the potential hepatotoxic components in Herba Epimedii caused liver injury by mechanisms involving oxidative stress and mitochondrial dysfunction [11,12,13]. Hence, when examining the hepatotoxic mechanism of the components from Herba Epimedii, exploring the mitochondrial pathway was a promising and attractive option.

In the present study, HepG2 cells were used to investigate apoptosis and the related mechanistic pathways in vitro. The main reason for choosing the HepG2 cell line was that these cells have limited drug-metabolizing enzymes, such as CYP450 and UGT, which can exclude the effect of cellular metabolism, provide a better view of the direct influence of the original forms of potential hepatotoxic components on apoptosis and help to reveal their difference before and after liver microsome metabolism. The results showed that sagittatoside A, baohuoside I and icaritin all caused significant apoptosis and produced a remarkable increase in the expression of Bax; moreover, the inhibition of Bcl-2 activity was observed directly in only the icaritin group (Figure 3). Further analysis showed that the ratio of Bax/Bcl-2 could be disturbed with increasing concentrations of sagittatoside A, baohuoside I and icaritin compared to the control group (Figure 4). Coupled with the upregulation of caspase-9 and caspase-3 (Figure 4) and the influences of ROS and MMP determined in previous research [11,12,13], it was clear that sagittatoside A, baohuoside I and icaritin could induce apoptosis and exert hepatotoxicity by regulating the intrinsic pathway. However, icariside I did not activate the initiator and executioner proteins of apoptosis to cause apoptosis, which also corresponded to the unchanged MMP [12]. Therefore, the mechanism by which icariside I causes liver injury might involve contributions from other pathways.

The liver is both an important target organ of toxic compounds and the main site of the metabolism of the flavonoid components of Herba Epimedii. Flavonoid components normally undergo phase I and II metabolism in the liver [23]. Phase I metabolism introduces polar groups onto compounds through reactions such as oxidation, hydrogenation, and hydrolysis, which are principally catalyzed by enzymes in the CYP450 family. Phase II metabolism involves binding between the inherent polar groups on the compound or those generated from phase I metabolism to the polar groups of endogenous small molecules through catalysis by UGTs, sulfate transferase (SULT), glutathione S-transferase (GST) and others. The flavonoid components in Herba Epimedii could undergo CYP450-catalyzed phase I metabolism to become hydrogenated or *O*-demethylated and undergo phase II metabolism via glucuronidation, sulfonation or combination with glutathione to generate metabolites with improved water solubility that can be absorbed or excreted [24]. Liver microsomes are mixed function oxidase systems with little interference from other factors, can be used with easily controlled reaction conditions and show quick performance and good reproducibility, and their use has become one of the most important methods in the field of drug metabolism research.

In this study, after incubation with human liver microsomes and initiating phase I and II metabolic reactions with the cytochrome CYP450 enzyme and UGT, the apoptosis caused by sagittatoside A, icariside I, baohuoside I and icaritin after metabolism was analyzed to evaluate the effects of metabolism. The results showed that after the metabolic response, the mixture of sagittatoside A or icaritin with liver microsomes could induce cell apoptosis, but baohuoside I could not; however, icariside I gained a new ability to induce apoptosis. To explain this phenomenon, the metabolic parameters and characteristics of sagittatoside A, icariside I, baohuoside I and icaritin in a variety of liver microsomes were determined. The ability of the hepatotoxic flavonoid components to induce apoptosis would be weakened if they were quickly metabolized and largely consumed. Among the studied compounds, by comparing the *T*_1/2_ values, amounts remaining after metabolism and CLint (liver, in vitro) values of the more robust phase of metabolism in human liver microsomes, it was determined that icariside I and baohuoside I were more quickly metabolized and more thoroughly eliminated; additionally, sagittatoside A and icaritin were less influenced by metabolic elimination. Therefore, it could be inferred that the original forms of sagittatoside A and icaritin were more likely to be retained, while more icariside I and baohuoside I were probably transformed to metabolites. In addition, the types of metabolism for each compound were identified. Greater elimination of sagittatoside A and icariside was observed in phase I than phase II (Figure 5A,B and Figure 6A,B and Table 1 and Table 3, while baohuoside I and icaritin displayed the opposite result (Figure 5C,D and Figure 6C,D and Table 1 and Table 3). Consequently, sagittatoside A and icariside I mainly underwent phase I metabolism, and baohuoside I and icaritin mainly underwent phase II metabolism. It was reported that the β-glucosidic bonds at the C-3 and C-7 positions of the flavonoid constituents of Herba Epimedii are easily hydrolyzed via enzyme catalysis, but α-L-rhamnose, which is directly linked to the aglycone at the C-3 position, is difficult to remove [25,26], which could explain the main type of metabolism of the studied components. Because sagittatoside A has a disaccharide at the C-3 position and is directly linked to an α-L-rhamnoside moiety (Figure 1B), only the glucosyl group in the disaccharide was easily hydrolyzed to produce the phase I metabolite. The β-glucosidic bond of icariside I is at the C-7 position (Figure 1C), so it can be easily hydrolyzed during phase I metabolism. Baohuoside I has a typical α-L-rhamnosidic bond at the C-3 position (Figure 1D), leading to difficult cleavage by hydrolysis, but the hydroxyl group at the C-7 position can form a conjugate with glucuronic acid, forming phase II metabolites [27]. The C-3 and C-7 positions of icaritin both have hydroxyl groups (Figure 1E); thus, icaritin can participate in phase II metabolism. Importantly, these four compounds are related in terms of their metabolic transformation; baohuoside I is a phase I metabolite of sagittatoside A, and icaritin is a phase I metabolite of icariside I and baohuoside I [28,29]. The influence of metabolism on the apoptosis ability of these compounds could be summarized as follows: by metabolism through oral Herba Epimedii, some sagittatoside A remained in its original form because less metabolic elimination occurred. Sagittatoside A was even transformed to metabolites (such as baohuoside I and icaritin) based on the main phase I metabolism and could still induce apoptosis; icariside I could be quickly transformed to phase I metabolites (such as icaritin) in large quantities to induce apoptosis, which the original form did not possess; baohuoside I lost the ability to induce apoptosis due to quick and large phase II metabolism; and icaritin could still induce apoptosis in the original form due to the lower metabolic degree. However, an important detail to note is that flavonoid constituents may be cleaved in the stomach and/or in the intestines by glycosidases and potentially by gut microflora. Additionally, the metabolism mediated by enterocytes in the intestines could also have an effect on these investigated components before liver metabolism. Therefore, the method of using liver microsomes in vitro for studying components of metabolism cannot provide a complete picture.

In terms of differences in in vitro metabolism between species, each component presented its own metabolic characteristics. However, according to the CLint (liver, in vitro) results, it was proposed that the values from beagle dog microsomes were more similar to those from human microsomes. Comparing the metabolic parameters of the investigated components in two kinds of rodents’ microsomes, the values from SD rats were closer to those from humans. Moreover, considering the experimental costs and the ease of obtaining experimental animals, beagle dogs and SD rats are recommended for use in in vivo metabolism research of the potential hepatotoxic components of Herba Epimedii. At last, the in vitro results of this study provide a reference for the corresponding in vivo study, although there might be some differences due to complex processes in vivo and interaction among the components. Therefore, based on this research, it is essential to carry out a more comprehensive study on the in vivo mechanism and metabolism of the hepatotoxic components of Herba Epimedii in the future, which will help to guide the reasonable utilization of this TCM in the clinic and reduce safety risks.

## 5. Conclusions

The goal of this study was to further explore the mechanism related to the apoptosis of potential hepatotoxic components from Herba Epimedii, including sagittatoside A, icariside I, baohuoside I and icaritin, in vitro and preliminarily evaluate the effect of their metabolic characteristics on their role in apoptosis. Among the studied components, the hepatotoxic mechanism of sagittatoside A, baohuoside I and icaritin involved regulating the key proteins of the intrinsic pathway of apoptosis. After metabolism, the four components illustrated (to different degrees) the changes in roles related to apoptosis induction. By determining and comparing their metabolic stability in different kinds of liver microsomes by HPLC–MS/MS, it was determined that sagittatoside A could induce apoptosis in its original form and phase I metabolite, icariside I could induce apoptosis in phase I metabolite, baohuoside I could lose its ability to induce apoptosis due to phase II metabolism, and icaritin experienced reduced metabolic elimination and could induce apoptosis in its original form. Although this study provides a reference for the exploration of the hepatotoxicity of Herba Epimedii, the limitations of the in vitro method should be noted, especially in view of the complex drug-metabolizing enzyme system in vivo. Moreover, given the complexity and comprehensiveness of the hepatotoxicity of Herba Epimedii, more in-depth clinical research in vivo will be our next important direction.

## Figures and Tables

**Table 1:** Metabolic parameters after phase I metabolism of the hepatotoxic components of Herba Epimedii in liver microsomes from different species.

Compound.	Species	−*k*	*T*_1/2_ (min)	CLint_(mic)_ (μL/min/mg Protein)	CLint_(liver, in vivo)_ (mL/min/kg)
Sagittatoside A	Mice	0.0228	30.39	114.00	75.04
	SD rats	0.0337	20.56	168.50	46.56
	Humans	0.0170	40.76	85.00	16.48
	Rhesus monkeys	0.0184	37.66	92.00	32.49
	Beagle dogs	0.0181	38.29	90.50	25.04
Icariside I	Mice	0.0380	18.24	190.00	80.38
	SD rats	0.0577	12.01	288.50	49.73
	Humans	0.0347	19.97	173.50	18.51
	Rhesus monkeys	0.0419	16.54	209.50	38.08
	Beagle dogs	0.0189	36.67	94.50	25.25
Baohuoside I	Mice	0.0186	37.26	93.00	72.33
	SD rats	0.0375	18.48	187.50	47.29
	Humans	0.0248	27.94	124.00	17.67
	Rhesus monkeys	0.0325	21.32	162.50	36.65
	Beagle dogs	0.0350	19.80	175.00	27.60
Icaritin	Mice	0.0200	34.65	100.00	73.33
	SD rats	0.0120	57.75	60.00	36.44
	Humans	0.0154	45.00	77.00	16.12
	Rhesus monkeys	0.0207	33.48	103.50	33.46
	Beagle dogs	0.0286	24.23	143.00	26.94

**Table 2:** Metabolic parameters after phase II metabolism of the hepatotoxic components of Herba Epimedii in liver microsomes from different species.

Compound	Species	−*k*	*T*_1/2_ (min)	CLint_(mic)_ (μL/min/mg Protein)	CLint_(liver, in vitro)_ (mL/min/kg)
Sagittatoside A	Mice	0.0151	45.89	75.50	69.18
	SD rats	0.0186	37.26	93.00	41.40
	Humans	0.0093	74.52	46.50	13.98
	Rhesus monkeys	0.0156	44.42	78.00	31.03
	Beagle dogs	0.0098	70.71	49.00	21.54
Icariside I	Mice	0.0336	20.63	168.00	79.28
	SD rats	0.0261	26.55	130.50	44.57
	Humans	0.0126	55.00	63.00	15.32
	Rhesus monkeys	0.0313	22.14	156.50	36.42
	Beagle dogs	0.0169	41.01	84.50	24.71
Baohuoside I	Mice	0.0334	20.75	167.00	79.22
	SD rats	0.0633	10.95	316.50	50.16
	Humans	0.0452	15.33	226.00	19.03
	Rhesus monkeys	0.0707	9.80	353.50	40.29
	Beagle dogs	0.0480	14.44	240.00	28.45
Icaritin	Mice	0.0378	18.33	189.00	80.34
	SD rats	0.0281	24.66	140.50	45.18
	Humans	0.0261	26.55	130.50	17.81
	Rhesus monkeys	0.0280	24.75	140.00	35.69
	Beagle dogs	0.0458	15.13	229.00	28.34

**Table 3:** Compound ion pairs and detection parameters.

Compound	Q1 Mass (Da)	Q3 Mass (Da)	Dwell Time (ms)	DP (volts)	EP (volts)	CE (volts)	CXP (volts)
Sagittatoside A	675.30	366.20	100	−22.90	−10.00	−48.00	−15.00
Icariside I	529.30	367.10	100	−14.70	−10.00	−33.30	−15.00
Baohuoside I	513.20	366.20	100	−12.50	−10.00	−37.50	−15.00
Icaritin	367.10	308.90	100	−12.80	−10.00	−36.70	−15.00

## Data Availability

Data are contained within the article.
